# Diagnostic Assessment of Periodontal and Dentoalveolar Complications Following Mini-Screw-Assisted Rapid Palatal Expansion in Adults and Late Adolescents: A Systematic Review

**DOI:** 10.3390/diagnostics16020352

**Published:** 2026-01-21

**Authors:** Barbara Frenna, Raffaella Grimaldi, Salvatore Fiandaca, Renisa Basha, Monica Caprio, Giacomo Emanuele Maria Rizzo, Alessio Verdecchia, Enrico Spinas

**Affiliations:** 1 Department of Surgical Sciences, Orthodontics School, University of Cagliari, 09124 Cagliari, Italy; barbarafre@outlook.it (B.F.); grimaldi.raff@gmail.com (R.G.); fiandaca92@gmail.com (S.F.); momocaprio@icloud.com (M.C.); 2 Faculty of Medicine and Surgery, School of Dentistry, Catholic University “Our Lady of Good Counsel”, 1000 Tirana, Albania; renisabasha@gmail.com; 3 Gastroenterology and Endoscopy Unit, La Maddalena Cancer Center, 90127 Palermo, Italy; 4 Orthodontics Division, Instituto Asturiano de Odontología, Universidad de Oviedo, 33006 Oviedo, Spain

**Keywords:** MARPE, mini-screw-assisted rapid palatal expander, diagnosis, CBCT, imaging, maxillary discrepancy, palatal expansion

## Abstract

**Objectives**: This systematic review aimed to evaluate the effectiveness of currently available methods for the diagnosis and monitoring of skeletal, dental, and soft tissue changes, as well as the adequacy of follow-up protocols, in adolescents and adults treated with miniscrew-assisted rapid palatal expansion (MARPE). **Materials and Methods**: This systematic review was conducted in accordance with the PRISMA guidelines. A comprehensive electronic literature search was performed across five databases (PubMed, Scopus, Embase, Cochrane, and Web of Science) to identify prospective and retrospective clinical studies evaluating dental, periodontal, and alveolar bone outcomes associated with MARPE in late adolescent and adult patients. Study selection, data extraction, and risk of bias assessment were independently performed by two reviewers. Risk of bias was assessed using the ROBINS-I tool for non-randomized studies and the RoB 2 tool for randomized studies. The certainty of the evidence was evaluated using the GRADE approach. Owing to substantial methodological heterogeneity and limited follow-up duration, a structured qualitative (narrative) synthesis of the results was performed. **Results**: A total of 20 studies were included in the systematic review. The reported adverse events primarily involved hard and soft tissues and were identified using cone-beam computed tomography (CBCT), clinical and periodontal examination, panoramic and cephalometric radiography, and digital dental casts. Dental effects, including dental tipping, were frequently reported across the included studies. Alveolar bone loss was reported in 11 studies, buccal alveolar bone dehiscence in 3 studies, and failure of palatal suture opening in 6 studies. In most of the included studies, follow-up was either not reported or limited. **Conclusions**: The MARPE technique appears to be potentially effective in achieving transverse maxillary expansion in late adolescent and adult patients. However, the included studies report possible adverse events affecting periodontal and alveolar bone tissues, such as alveolar bone thinning and gingival hypertrophy, the assessment of which requires an integrated diagnostic approach combining CBCT imaging with clinical and periodontal examination. Overall, the certainty of the available evidence was low to very low, mainly due to a high risk of bias, methodological heterogeneity, and limited or absent follow-up in most studies. Therefore, the results should be interpreted with caution. Well-designed prospective controlled studies with standardized protocols and long-term follow-up are needed to conclusively evaluate the safety and long-term clinical stability of the MARPE technique.

## 1. Introduction

Transverse maxillary discrepancy is a serious problem that every orthodontist must be able to identify and diagnose. In the past, only pediatric patients were treated.

Nowadays, thanks to newly available devices, the range of patients has been extended to include adults [1].

Upper jaw expansion is a highly important orthodontic procedure with a high success rate, which largely depends on the appropriate choice of treatment and the age of the patient [2]. Transverse maxillary deficiency is a common clinical condition that can cause severe malocclusions, such as dental crowding and crossbites, compromising both the patient’s oral function and esthetics [3]. 

One of the most established methods is Rapid Palatal Expansion (RPE), a successful orthodontic technique for maxillary expansion and correction of transverse discrepancies, especially in children. This technique is based on the separation of the midpalatal suture, which occurs when a rapid transverse force is applied to the maxillary teeth, promoting bone remodeling along the sutural areas and facilitating skeletal expansion. 

Conventional RPE maxillary expansion has limited skeletal effects due to increased interdigitation of the midpalatal suture and adjacent joints [4]. Traditional RPE can also cause adverse dento-skeletal events, gingival recession, fenestrations, and root resorption of the supporting teeth [5]. 

In adults, the likelihood of complete maturation of the midpalatal suture is significantly higher, meaning that greater stress is required to open the circummaxillary and midpalatal sutures. Therefore, RPE is less effective in adults. For this reason, alternative techniques have been developed for adults, such as MARPE (Mini-Implant-Assisted Rapid Palatal Expander), which allows transverse forces to be applied directly to the palate using orthodontic mini-implants [6]. 

MARPE produces more significant skeletal effects and reduces the periodontal and dental side effects that would occur if expansion in adult patients had been performed using traditional expansion [7]. Nowadays, the use of mini-implants in orthodontics is becoming increasingly widespread, which has expanded the possibilities for direct anchorage of expansion appliances to the palatal bone, improving the effectiveness and safety of disjunction procedures [8]. 

Although this technique achieves significant skeletal expansion with reduced surgical morbidity, several periodontal and dentoalveolar complications have been reported, including dental tipping, buccal alveolar bone dehiscence, gingival hypertrophy, and failure of mid-palatal suture opening. Accurate diagnosis and monitoring of these adverse events are crucial for ensuring treatment safety and long-term stability. 

In this context, diagnostic tools such as cone-beam computed tomography (CBCT), conventional radiography, and clinical or periodontal examinations play a central role in detecting morphological and soft-tissue changes induced by MARPE [9]. 

Overall, MARPE can be considered an effective solution for correcting transverse maxillary discrepancies in non-growing patients, offering more stable skeletal expansion with fewer side effects than traditional methods [10,11].

Therefore, the primary aim of this systematic review was to assess the safety of MARPE in late adolescents and adults by synthesizing evidence on the type and frequency of adverse events. A secondary aim was to evaluate the diagnostic methods used to detect and monitor periodontal, dentoalveolar, and skeletal complications.

## 2. Materials and Methods

### 2.1. Search Strategy

This study aimed to identify and evaluate the safety of MARPE among adult patients with maxillary discrepancy [12,13,14]. The study followed the Preferred Reporting Items for Systematic Reviews and Meta-Analyses (PRISMA) statement [15], and the protocol was registered in the Prospective Register of Systematic Reviews (PROSPERO) with the registration number CRD420251036286 (Table S1). The primary research question was: “Is the mini-screw-assisted rapid palatal expander (MARPE) safe in the treatment of maxillary discrepancy in adult patients?”, which was used as clinical question in order to follow the PICO framework. Accordingly, the following items were defined: (P) adult patients and late adolescents (older than 16 years) with maxillary discrepancy, as the midpalatal suture presents a high level of maturity that makes its expansion more difficult to achieve; (I) MARPE; (C) no treatment; (O) safety (rate of adverse events [AEs]). Combinations of the following Medical Subject Headings (MeSH) terms were used: ‘MARPE’, ‘miniscrew’, ‘expanders’, ‘palatal’, and ‘efficacy’. The detailed search strategy is presented in Table 1. A comprehensive search was conducted up to July 2025. The primary sources of the reviewed studies were PubMed, Web of Science, Embase, EBSCO, Cochrane, and Scopus. The research was conducted without temporal or language restrictions to ensure the retrieval of all available scientific information. To identify additional studies, the computer search was supplemented with manual searches of the reference lists of the retrieved reviews and studies.

### 2.2. Eligibility Criteria

#### 2.2.1. Inclusion Criteria

Studies were included if they (1) included adults and late adolescents with transverse maxillary deficiency treated with MARPE; (2) reported at least one pre-specified outcome of interest, including adverse events (AEs) related to dental, periodontal, osseous, soft tissue, or device-related complications, or treatment success; (3) were randomized or non-randomized clinical trials or observational studies, either prospective or retrospective.

#### 2.2.2. Exclusion Criteria

Studies were excluded if they (1) included pediatric patients (under 16 years of age), patients with cleft lip and palate or craniofacial anomalies; (2) included patients with a history of maxillofacial surgery; (3) were case reports or case series, due to their limited methodological robustness for safety assessment; or (4) were available only in abstract form.

### 2.3. Data Extraction

Two reviewers (B.F. and R.G.) independently extracted outcome data from the included studies and subsequently screened the collected results for accuracy and completeness. For each included study, the following data were extracted: first author and year of publication; country in which the study was conducted; study design; sample characteristics (including age, sex, and ethnicity, when available); technical characteristics of the MARPE procedure; and the number and type of reported adverse events (AEs). All studies were independently assessed and classified by two reviewers (B.F. and R.G.). Disagreements in qualitative or quantitative data extraction were infrequent (overall interobserver variability <10%) and were resolved through discussion. When consensus could not be reached, a third reviewer (E.S.) was consulted for arbitration. Adverse events were extracted and classified according to the pre-specified definitions and categories described below.

### 2.4. Definition and Classification of Adverse Events

In this systematic review, the term “adverse events (AEs)” is used consistently to refer to all clinically relevant complications associated with MARPE. In consideration of the heterogeneity of the included studies and the lack of a standardized definition of adverse events (AEs) in the miniscrew-assisted rapid palatal expansion (MARPE) literature, adverse events were defined and classified a priori, before study selection and data extraction, based on their clinical and biological relevance, in order to ensure consistency and reduce the risk of selective outcome reporting. It is essential to distinguish between physiologic remodeling and pathological complications. While many studies report a 100% rate of ‘adverse events’, these often include expected biological responses such as transient gingival inflammation or mild buccal bone thinning. In this review, clinically significant adverse events were defined as outcomes that compromise long-term periodontal health, such as severe bone dehiscence or loss of miniscrew stability. Based on these pre-specified definitions, AEs were classified into the following categories:

1. Osseous and dentoalveolar events, including alveolar bone loss, reduction in buccal alveolar bone thickness, and buccal alveolar bone dehiscence;
2. Periodontal and gingival events, such as gingival hypertrophy or clinically relevant gingival inflammation;
3. Failure of expansion, defined as failure of mid-palatal suture opening;
4. Device-related adverse events, including miniscrew loosening or failure, fracture or deformation of the expansion device, soft tissue irritation related to the appliance, or other device-related complications, when explicitly reported in the included studies.

### 2.5. Quality Assessment

The risk of bias of included randomized controlled trials was assessed using version 2 of the Cochrane Risk of Bias tool (RoB 2) [16]. Non-randomized studies of interventions were evaluated using the Risk of Bias in Non-randomized Studies of Interventions tool (ROBINS-I) [17]. All assessments were conducted independently by two reviewers, with disagreements resolved through discussion or consultation with a third reviewer when necessary. For non-randomized studies, the risk of bias was assessed across the seven ROBINS-I domains:

- Bias due to confounding, evaluated by examining whether patient characteristics or clinical factors could have influenced the occurrence or reporting of adverse events (AEs);
- Bias in selection of participants, assessed by reviewing recruitment methods and exclusion criteria and their potential impact on sample representativeness;
- Bias in classification of interventions, evaluated by verifying the accurate distinction of MARPE from other maxillary expansion techniques;
- Bias due to deviations from intended interventions, assessed by analyzing adherence to activation protocols and treatment procedures;
- Bias due to missing data, evaluated by reviewing the completeness of follow-up and the reporting of AEs;
- Bias in measurement of outcomes, assessed by considering the reliability, objectivity, and consistency of AE assessment methods across studies;
- Bias in selection of the reported result, evaluated by comparing reported outcomes with study protocols or expected AE profiles, when available.

Each study was categorized as having low, moderate, or serious risk of bias according to the criteria of the respective assessment tools. The certainty of the evidence for the primary adverse event outcomes was evaluated using the GRADE (Grading of Recommendations, Assessment, Development and Evaluation) approach [18]. The following domains were considered: risk of bias, inconsistency, indirectness, imprecision, and publication bias. Downgrading of the certainty of evidence was primarily driven by the high or serious risk of bias observed in the included studies, heterogeneity in outcome definitions and measurement methods, small sample sizes, and the lack of long-term follow-up data. Based on these considerations, the overall certainty of the evidence for the primary safety outcomes was judged to be low to very low. Secondary and less frequently reported adverse events were summarized descriptively in tables but were not included in the GRADE assessment, as they were inconsistently reported and not sufficiently homogeneous to allow a reliable certainty assessment.

### 2.6. Data Synthesis and Management of Heterogeneity

Due to substantial clinical and methodological heterogeneity across the included studies—including differences in study design, diagnostic methods, expansion protocols, follow-up duration, and outcome reporting—a quantitative meta-analysis was not deemed appropriate. A structured narrative synthesis was therefore performed. Studies were grouped and analyzed according to the pre-specified categories of adverse events (osseous, periodontal and gingival, failure of expansion, and device-related events). Within each category, results were further examined in relation to study design and follow-up duration when feasible. This approach allowed for a systematic comparison of patterns, consistency, and clinical relevance of adverse events across studies, while explicitly accounting for heterogeneity in study characteristics and outcome definitions.

## 3. Results

**Figure 1:**
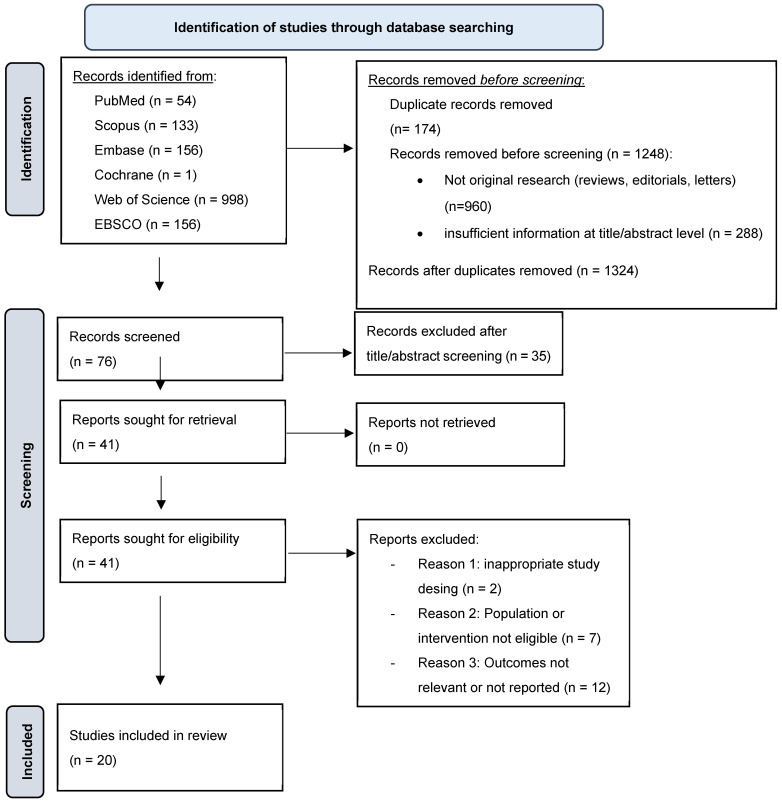
PRISMA flow diagram illustrating the study selection process.

The electronic database search identified a total of 1498 records, including PubMed (n = 54), Scopus (n = 133), Embase (n = 156), the Cochrane Library (n = 1), Web of Science (n = 998), and EBSCO (n = 156). After removal of 174 duplicate records, 1324 records remained. Of these, 1248 records were excluded prior to screening because they were not original research articles (e.g., reviews, editorials, letters to the editor) or because they contained insufficient information at the title and abstract level to assess eligibility. The remaining 76 records were screened based on titles and abstracts, and 35 records were excluded as they did not meet the predefined inclusion criteria. Consequently, 41 full-text articles were assessed for eligibility. Of these, 21 articles were excluded for the following reasons: inappropriate study design (n = 2), population or intervention not eligible (n = 7), and outcomes not relevant or not reported (n = 12). Finally, 20 studies were included in the qualitative synthesis (Figure 1). These comprised 2 randomized controlled trials, 5 prospective clinical studies, and 13 retrospective studies.

### 3.1. Description of the Included Studies

Most of the included studies were conducted outside Europe, primarily in Asia. Overall, 758 patients undergoing MARPE were included. The proportion of male participants varied widely among studies, and the highest reported mean age was 36.85 years. Dental tipping was frequently reported across the included studies; however, it was considered an expected biomechanical consequence of the expansion mechanics rather than a pathological adverse event and was therefore excluded from the summary tables and from the calculation of overall adverse event (AE) rates. Accordingly, the reported AE rates, which ranged from 62.5% to 100%, reflect only clinically relevant adverse events. This range reflects differences in reporting across the included studies: some studies explicitly reported the proportion of patients experiencing adverse events, whereas others described adverse events as occurring in all treated patients without providing numerical estimates and were therefore considered as reporting a 100% adverse event rate. For several included studies, adverse events were reported descriptively without separate quantification by category; therefore, outcome-level data could not be extracted and are reported as N/R in Table 2 and Table 3. Follow-up was not reported or was insufficient in the majority of studies (n = 19). 

Regarding appliance design, all included studies used a single, specific type of expander. A tooth–bone-borne design was employed in 17 studies, whereas a bone-borne design was used in 3 studies. The use of a Hyrax-type expander was reported or could be deduced in 16 studies. Among the studies employing a tooth–bone-borne design, the use of a maxillary skeletal expander (MSE) was explicitly reported in 4 studies (23.5%). Clinically relevant adverse events were classified into osseous, periodontal/gingival, expansion failure, and device-related events. Reported adverse events included buccal alveolar bone dehiscence, failure of mid-palatal suture opening, and gingival alterations, as well as isolated device-related complications, such as miniscrew migration associated with pressure ulcers. A notable finding was the correlation between patient age and the failure of mid-palatal suture opening. While MARPE is designed for non-growing patients, the increased interdigitation of the circummaxillary sutures in older adults remains a primary limiting factor. In the reviewed studies, expansion failure was more frequently observed in patients approaching the third decade of life, suggesting that chronological age remains a critical predictor of skeletal success. As noted, the limited or absent follow-up in most studies restricts conclusions regarding the long-term incidence and clinical significance of these adverse events.

A summary of the characteristics is reported in Table 2 and Table 3.

### 3.2. Diagnostic Methods and Outcome Assessment

The included studies adopted heterogeneous diagnostic approaches to evaluate skeletal and dental changes after MARPE. Most studies (n = 16) employed cone-beam computed tomography (CBCT) to assess skeletal expansion and alveolar bone response, while 2 studies used two-dimensional radiographs or dental casts for transverse measurements. Digital model analysis was applied in 1 study. CBCT imaging emerged as the gold standard for monitoring MARPE outcomes, being used in 80% of the included studies. Unlike 2D radiography, CBCT allows for the precise quantification of cortical bone thickness and the detection of buccal bone dehiscence that might otherwise be masked by dental tipping. Future research should prioritize standardized CBCT protocols to ensure comparability across clinical trials. A summary of the diagnostic methods and measurement parameters is provided in Table 4.

### 3.3. Synthesis of Key Domains

#### 3.3.1. Adverse Events (AEs)

Clinically relevant adverse events were reported in all included studies, with reported AE rates ranging from 62.5% to 100%. Alveolar bone loss or thinning was reported in 11 of the 20 included studies, involving approximately 758 patients. Buccal alveolar bone dehiscence was reported in 3 studies, while failure of mid-palatal suture opening was reported in 6 studies. 

A structured descriptive summary of the main clinically relevant adverse outcomes, including the number of studies reporting each event and the reported ranges or proportions, is provided in Table 5.

#### 3.3.2. Diagnostic Tools

Most studies assessed outcomes using cone-beam computed tomography (CBCT), while a smaller number relied on two-dimensional radiographs or digital dental casts.

#### 3.3.3. Follow-Up

Follow-up duration was not reported or was limited in the majority of the included studies.

#### 3.3.4. Expansion Protocol

Activation protocols varied across studies, ranging from quarter-turn activation regimens to more aggressive daily activation schedules. Retention protocols were inconsistently reported.

#### 3.3.5. Type of Appliance

Most studies investigated tooth–bone-borne expanders, including devices such as the maxillary skeletal expander (MSE), whereas only a limited number evaluated purely bone-borne appliances.

### 3.4. Technical Findings

The expansion protocols among the included studies were notably heterogeneous, limiting direct comparisons. Activation strategies varied: nearly half of the studies used a 1/4-turn activation, either once or multiple times daily, while others used a full turn per day or more aggressive protocols of two turns daily. Similarly, the synthesis of technical findings revealed a significant lack of standardization regarding activation rates, ranging from conservative schedules (0.2 mm every other day) to aggressive schedules (2 turns per day). Retention periods also varied considerably, from two to twelve months, with several studies not reporting this information at all. This variability highlights the absence of standardized MARPE protocols for adult patients and represents a methodological limitation affecting reproducibility, interpretation of outcomes, and the establishment of a definitive ‘safe’ activation rate, underscoring a crucial gap in the current orthodontic literature regarding optimal force-delivery systems.

### 3.5. Risk of Bias Assessment

The complete risk of bias assessments using the RoB 2 and ROBINS-I tools are presented in Table 6 and Table 7, respectively, summarizing the judgments across all relevant domains for each included study. Two studies were randomized controlled trials and were assessed using the RoB 2 tool; both were judged to have a moderate risk of bias. The remaining 18 studies were non-randomized studies of interventions and were evaluated using the ROBINS-I tool. Among these, three studies were judged to have a serious risk of bias, while the remaining studies were assessed as having a moderate risk of bias. No study was classified as having a low risk of bias according to the ROBINS-I criteria (Table 8). Adverse events that were less frequently reported or inconsistently assessed across studies were summarized narratively and presented in descriptive tables. These outcomes were not included in the GRADE assessment, which was restricted to the most clinically relevant and consistently reported adverse events.

The GRADE analysis is shown in Table 9.

According to the GRADE approach, the overall certainty of evidence for primary safety outcomes was judged as low to very low. This downgrading was primarily due to the predominance of retrospective study designs, the lack of long-term follow-up, and the high risk of bias related to inconsistent reporting of minor complications across the included literature. Consequently, the clinical recommendations derived from these findings should be applied with caution.

## 4. Discussion

The effectiveness of MARPE appears to be influenced by multiple factors, contributing to the complexity of interpreting the available evidence. The present systematic review analyzed studies that suggest the ability of MARPE to induce skeletal expansion in both late adolescents and adults [19,20,24,39]. However, the reported outcomes show considerable variability, which seems to be associated with differences in patient age, appliance design, and clinical protocols. These observations are consistent with the findings of the systematic review and meta-analysis by Kapetanović et al. (2021) [8], in which the authors concluded that MARPE can be associated with significant skeletal expansion in late adolescents and adults, while also reporting substantial heterogeneity among the included studies. Their results highlight how variations in age, appliance design, and study methodology may significantly influence treatment outcomes, reinforcing the need for standardized clinical protocols [8]. Although MARPE is generally proposed for the treatment of transverse maxillary deficiency [40] in adult patients, its use was also explored in late adolescents and young adults in the present review. A total of 20 studies, including 758 patients, were analyzed. Overall, the included studies reported high clinical effectiveness, although long-term follow-up was frequently limited or absent. The safety of MARPE is generally attributed to the use of miniscrews, which provide enhanced appliance stability [40], and the incidence of serious adverse events (AEs) appears to be very low [8]. The relatively high rates of reported adverse events should be interpreted with caution, as they reflect substantial heterogeneity in outcome definitions, diagnostic methods, and reporting practices across studies, rather than a high incidence of severe clinical complications. However, dental tipping may be more appropriately interpreted as a mild and expected biomechanical consequence of force distribution related to the appliance design rather than a true clinical complication. The diagnosis of periodontal and dentoalveolar complications following MARPE is typically based on a combination of imaging and clinical assessment tools, each with distinct advantages and limitations. Cone-beam computed tomography (CBCT) represents the most comprehensive diagnostic modality, allowing three-dimensional visualization of skeletal and alveolar structures. CBCT enables detailed assessment of buccal cortical bone thickness, alveolar dehiscence, and root proximity—findings that are often underestimated using conventional two-dimensional radiography. Nevertheless, its diagnostic use should be critically balanced against concerns related to radiation exposure, cost, and variability in image interpretation across studies. Conventional radiography remains a valuable adjunctive diagnostic tool due to its accessibility and relatively low radiation dose. However, inherent limitations related to projection errors and superimposition of anatomical structures may result in underestimation of bone loss or dental tipping. Consequently, while conventional radiographs are suitable for initial screening and routine post-treatment follow-up, their integration with CBCT may be indicated when a detailed evaluation of alveolar integrity is required. Clinical and periodontal examinations constitute essential diagnostic approaches for identifying soft-tissue changes, such as gingival hypertrophy, recession, or inflammation. These assessments are non-invasive and repeatable, allowing longitudinal monitoring throughout treatment. However, they may lack sensitivity in detecting early subclinical alterations of the alveolar bone. The integration of clinical findings with imaging modalities appears to enhance diagnostic reliability and provides a more comprehensive evaluation of potential MARPE-related complications. Overall, the combined use of CBCT, conventional radiography, and clinical-periodontal assessment allows for a multidimensional diagnostic approach, which may improve the early detection and management of adverse events. Nevertheless, future research should aim to standardize diagnostic protocols and establish shared quantitative criteria for interpreting post-MARPE changes, thereby improving reproducibility and supporting clinical decision-making. In younger patients, rapid maxillary expansion has been associated with increases in nasal cavity dimensions and improvements in nasal respiratory patterns, as reported in studies using acoustic rhinometry and computed rhinomanometry [41]. Although MARPE may represent an alternative to surgically assisted rapid palatal expansion (SARPE), its indication should be carefully evaluated, taking into account individual anatomical characteristics and treatment objectives [42]. Several studies suggest that MARPE may induce a greater degree of rotation and tilting of the maxillary segments compared with other expansion techniques [43]. In particular, Byloff et al. [44] reported that skeletal expansion achieved with SARPE primarily consists of lateral rotation of the maxillary hemiarches, with limited horizontal translation. While SARPE generally offers higher predictability and stability in cases of severe transverse discrepancies, it is more invasive, requires surgical intervention by maxillofacial surgeons, and is associated with higher costs and morbidity compared with MARPE [42]. Nevertheless, SARPE remains an appropriate option in cases of advanced palatal suture ossification or when the skeletal expansion achievable with MARPE is insufficient. Therefore, the choice between MARPE and SARPE should be based on an individualized assessment of anatomical characteristics, severity of the transverse discrepancy, and patient preferences. Based on the available data, MARPE appears to be associated with favorable skeletal modifications beyond the pediatric age range, although its effectiveness may decrease with increasing age. In the included studies, the highest reported mean age was 36.85 years, suggesting that older patients may not experience the same benefits observed in younger adults, as also noted by Sicca et al. [45]. The long-term stability of MARPE-induced expansion remains incompletely understood. While some studies report encouraging long-term outcomes [19,20], relapse or recurrence has not been systematically investigated, largely due to limited follow-up durations or the absence of studies specifically designed to evaluate stability. Appliance design also appears to play a critical role in treatment outcomes. Bone-borne MARPE appliances, anchored directly to the palatal bone, may be associated with greater skeletal expansion and fewer adverse events compared with tooth-borne devices [37,45]. In addition, the position and characteristics of miniscrews may influence the biomechanical forces generated during expansion and the resulting skeletal response. In a randomized controlled trial, Choi et al. reported that MARPE using longer miniscrews was associated with a greater degree of maxillary basal bone and canine alveolar bone expansion [31]. Considerable heterogeneity was also observed in activation protocols and retention periods, which ranged from 2 to 12 months across the included studies. Most studies reported quarter-turn activations at least once daily, whereas others applied one or two full turns per day, highlighting the operator-dependent nature of MARPE protocols. Ultimately, treatment decisions should be individualized, taking into account desired clinical outcomes, anatomical constraints, and patient preferences, particularly when both MARPE and SARPE are viable treatment options [46]. Although the present review adds to the growing body of evidence supporting the use of MARPE in late adolescent and adult populations, the findings must be interpreted in light of several important limitations. The included studies exhibited substantial methodological heterogeneity in terms of study design, sample size, reported outcomes, and statistical analyses. Many studies lacked control groups, randomization, or blinding, increasing the risk of bias. The predominance of retrospective and observational designs limits internal validity and reduces the strength of conclusions that can be drawn from the aggregated data. An important additional limitation is the generally short or absent follow-up in most included studies, which substantially limits the ability to detect late-onset adverse events, particularly progressive periodontal or alveolar bone changes that may emerge over time. Finally, the risk of bias was relevant across several domains, particularly selection bias and the lack of measures addressing performance and detection bias. Given the relatively early stage of MARPE research and the generally limited sample sizes, large-scale, prospective, well-controlled studies with standardized protocols, uniform adverse event definitions, and long-term follow-up are required to provide more robust and clinically reliable evidence. Furthermore, a significant portion of the evidence base originates from Asian populations. This geographical clustering may limit the generalizability of the results, as ethnic variations in craniofacial morphology and sutural maturation patterns could influence the threshold for skeletal expansion and the susceptibility to periodontal adverse events in other populations.

## 5. Conclusions

This systematic review indicates that MARPE treatment is potentially effective in achieving transverse maxillary expansion in selected late adolescent and adult patients. Expected dental effects, such as dental tipping, appear to represent intrinsic biomechanical consequences of the expansion process rather than pathological adverse events. Nevertheless, MARPE may be associated with clinically relevant adverse outcomes affecting periodontal and alveolar bone tissues, including alveolar bone thinning, dehiscence, and gingival alterations.

Accurate identification of these adverse events requires a comprehensive diagnostic strategy. Cone-beam computed tomography (CBCT) appears to provide higher diagnostic accuracy for detecting alveolar bone changes, whereas clinical and periodontal examinations remain essential for identifying soft-tissue alterations, such as gingival hypertrophy. Accordingly, an integrated diagnostic approach combining three-dimensional imaging with clinical and periodontal assessment may allow a more complete evaluation of treatment-related changes and support early detection and prevention of MARPE-associated complications in both clinical and research settings. However, the limited number of available studies, substantial methodological heterogeneity, high risk of bias, and the frequent absence or short duration of follow-up considerably reduce the certainty of the available evidence, which was judged to be low to very low. Consequently, the present findings should be interpreted with caution. Further well-designed prospective studies with standardized diagnostic protocols, adequate sample sizes, and long-term follow-up are needed to clarify the long-term periodontal and skeletal effects of MARPE, identify predictors of adverse outcomes, and strengthen the overall quality of the evidence.

## Figures and Tables

**Table 1:** Search strategy divided for each database.

PubMed	((MARPE)) OR (palatal expander) OR (palatal expansion)) AND ((miniscrews)) e ((adverse events) OR (efficacy))
Scopus	(“palatal expansion”) AND (“miniscrew” OR “orthodontic mini implant” OR “palatal miniscrew”) AND (“side effect” OR (“adverse” AND “effect”)) AND (“adult” OR “late adolescents”) AND (“orthodontics”) AND (“MARPE” OR “palatal expander”))
Embase	((‘palatal expansion’/exp OR ‘palatal expansion’) AND (‘miniscrew’ OR ‘orthodontic mini implant’ OR ‘palatal miniscrew’) AND (‘side effect’ OR (adverse AND effect)) AND (‘adult’ OR ‘late adolescence’) AND ‘orthodontics’)
Cochrane	((“palatal expansion”) AND (“miniscrew”) OR “orthodontic mini implant” OR “palatal miniscrew”) AND (“side effect” OR (“adverse” AND “effect”)) AND (“adult” OR “late adolescence”) AND (“orthodontics”) AND (“MARPE” OR “palatal expander”))
EBSCO	(“MARPE”) AND (“PALATAL EXPANDER”)
Web of Science	“miniscrews” (All Fields) AND “MARPE” (All Fields) AND “palatal expander” (All Fields) AND “adverse effects” (All Fields) AND “adults” (All Fields)

**Table 2:** Study characteristics.

Study, Year	Design	Geographical Area	Patients, n	Age, Years	Gender, M (%)
Choi, 2016 [19]	Prospective, study	Republic of Korea	69	20.9 ± 2.9	10 (14.5)
Lim, 2017 [20]	Prospective, study	Republic of Korea	24	21.6	8 (33.3)
Bud ES, 2021 [21]	Observational Study	Romania	27	24	9 (33.3)
Madeeh S, 2023 [22]	Prospective study	Iraq	24	19.5 ± 2.5	12 (50)
Marin CM, 2023 [23]	Prospective, study	Brazil	19	24.92	5 (31.2)
Park, 2017 [24]	Retrospective, study	Republic of Korea	14 **	20.1 ± 2.4	9 (64.3)
Clement, 2017 [25]	Prospective study	India	10	21.5 ± 2.5	5 (50)
Ngan, 2018 [26]	Prospective, study	USA	8	21.9 ± 1.5	6 (75)
Shin, 2019 [27]	Retrospective, study	Republic of Korea	31	22.5 ± 5.11	16 (51)
Annarumma F, 2021 [28]	Retrospective, study	Italy	24	16	12 (50)
* Lee, 2020 [29]	Prospective study	Republic of Korea	30	N/R	N/R
Basu, 2023 [30]	Randomized clinical trial study	India	18	20.77	N/R
Choi, 2023 [31]	RCT	Republic of Korea	32	19–35 ^§^	N/R
Wang, 2023 [32]	Observational study	China	40	18–28 ^§^	25 (62.5)
Gokturk M, 2024 [33]	Retrospective, study	Turkey	30	16.82 ± 0.48	13 (43.3)
Kapetanovic A, 2022 [34]	Prospective study	Netherlands	34	27 ± 9.4	8 (23.5)
Calil, 2020 [35]	Comparative, study	Paraguay	37	24.92	N/R
Nevada R, 2023 [36]	Prospective study	Brazil	28	28.8	9 (32.15)
Ning R, 2023 [37]	Prospective, study	China	91	20.5 ± 4.5	N/R
Yoon A, 2022 [38]	Retrospective, study	USA	256	18.8 ± 8.6	130 (50.7)

^§^ range; * 20 patients were included in the study; ** 14 patients were included in the study. RCT = Randomized controlled clinical trial.

**Table 3:** Adverse events (AEs).

Study	Patients, n	Type of Expander	AEs	Types of Adverse Events	Folllow-Up
Choi, 2016 [19]	69	Tooth–bone-born; Hyrax-type	100%	N/R	N/R
Lim, 2017 [20]	24	Tooth–bone-born; Hyrax-type	100%	Buccal alveolar bone dehiscence	1 year
Bud ES, 2021 [21]	27	Tooth–bone-borne (MSE)	62.5%	Failure of opening of the mid-palatal suture; gingival hypertrophy; decrease in buccal and palatal bone levels	N/R
Madeeh S, 2023 [22]	24	Tooth–bone-borne (MSE)	100%	Failure of opening of the mid-palatal suture	N/R
Marin CM, 2023 [23]	19	Tooth–bone-born; Hyrax-type	100%	Failure of opening of the mid-palatal suture; reduction in vestibular bone thickness	N/R
Park, 2017 [24]	14	Bone-born; Hyrax-type	61%	Decrease in buccal bone thickness and crest height	N/R
Clement, 2017 [25]	10	Tooth–bone-borne (MSE)	48%	N/R	N/R
Ngan, 2018 [26]	8	Tooth–bone-borne (MSE)	100%	Reduction in buccal bone thickness	N/R
Shin, 2019 [27]	31	Tooth–bone-born; Hyrax-type	100%	Buccal alveolar bone dehiscence	N/R
Annarumma F, 2021 [28]	24	Bone-born; Hyrax-type	100%	Failure of opening of mid-palatal	N/R
Lee, 2020 [29]	30	Tooth–bone-born; Hyrax-type	100%	Soft tissue changes; nose widening and movement forward/downward	N/R
Basu, 2023 [30]	18	Bone-born; Hyrax-type	100%	Bone resorption	N/R
Choi, 2023 [31]	32	Tooth-born; Hyrax-type	100%	Failure of opening of the mid-palatal suture	N/R
Wang, 2023 [32]	40	Tooth-born; Hyrax-type	100%	Buccal alveolar bone dehiscence	N/R
Gokturk M, 2024 [33]	30	Tooth-born; Hyrax-type	90%	N/R	N/R
Kapetanovic A, 2022 [34]	34	Tooth-born; Dutch maxillary expansion device (D-MED)	100%	Decrease in buccal bone thickness; failure of opening of the mid-palatal suture	N/R
Calil, 2020 [35]	37	Tooth-born; Hyrax-type	100%	Loss of buccal bone	N/R
Nevada R, 2023 [36]	28	Tooth-born; Hyrax-type	100%	Decrease in buccal bone thickness	N/R
Ning R, 2023 [37]	91	Tooth-born; Hyrax-type	100%	Alveolar bone loss	N/R
Yoon A, 2022 [38]	256	Tooth-born; Hyrax-type	83.9%	Gingival inflammation	N/R

N/R = indicates that the adverse event was not separately reported or could not be reliably extracted from the original publication. In several studies, adverse events were described narratively or aggregated without distinction among specific categories, precluding outcome-level data extraction. Footnote: The terminology used to describe adverse events reflects that reported in the original studies; for consistency, all clinically relevant outcomes are referred to as adverse events (AEs) throughout the text.

**Table 4:** Diagnostic methods and outcome assessment of the included studies.

Study (Author, Year)	Diagnostic Method	Imaging Modality	Measurement Type	Timing of Evaluation
Choi, 2016 [19]	Prospective study	CBCT	Suture opening; skeletal/dental expansion; alveolar bending; molar tipping; stability	T1–T3 (pre, post, 1-year)
Lim, 2017 [20]	Prospective clinical study	CBCT, casts	Suture opening; skeletal expansion; dental tipping; alveolar changes; stability	T1–T3 (pre, post, 6–12 mo)
Bud ES, 2021 [21]	Observational study	CBCT, casts, clinical	Dental tipping; alveolar changes; skeletal expansion; complications	T1–T2 (pre, post)
Madeeh S, 2023 [22]	Prospective study	CBCT	Dental tipping; transverse movement; side effects	T1–T2
Marin CM, 2023 [23]	Prospective study	CBCT	Skeletal expansion; suture opening; tipping; age correlation	T1–T2
Park, 2017 [24]	Prospective clinical study	CBCT	Suture opening; skeletal expansion; tipping; alveolar movement	T1–T2
Clement, 2017 [25]	Prospective clinical study	CBCT	Suture opening; skeletal expansion; tipping; alveolar bone changes	T1–T2
Ngan, 2018 [26]	Pilot clinical study	CBCT, periodontal exam	Suture opening; skeletal/dental expansion; alveolar thickness; periodontal changes	T1–T2
Shin, 2019 [27]	Observational clinical study	CBCT	Suture opening; skeletal expansion; tipping; cortical thickness; predictors	T1–T2
Annarumma F, 2021 [28]	Prospective comparative clinical study	CBCT	Suture opening; skeletal/dental expansion; tipping; alveolar changes; age comparison	T1–T2
Lee, 2020 [29]	Prospective clinical	3D stereophotogrammetry (+CBCT ref.)	Nasal width; alar widening; nasal volume	T1–T2 (short-terms)
Basu, 2023 [30]	Randomized clinical trial	CBCT	Suture opening; skeletal/dental expansion; tipping; corticopunture effect	T1–T2
Choi, 2023 [31]	Randomized clinical trial	CBCT	Suture opening; skeletal/dental expansion; tipping; miniscrew length effect	T1–T2
Wang, 2023 [32]	Prospective clinical study	CBCT	Suture opening; skeletal/dental expansion; alveolar changes; sagittal-type comparison	T1–T2
Gokturk M, 2024 [33]	Prospective comparative clinical study	CBCT	Suture opening; skeletal/dental expansion; tipping; alveolar changes; appliance comparison	T1–T2
Kapetanovic A, 2022 [34]	Prospective clinical cohort study	CBCT + intra-oral scans (IOS)	Skeletal expansion; alveolar expansion; dental expansion; buccal bone thinning; clinical crown height; periodontal side-effects	T1–T2
Calil, 2020 [35]	Prospective clinical study	CBCT	Suture opening; skeletal/dental expansion; tipping; alveolar changes	T1–T2
Nevada R, 2023 [36]	Prospective comparative clinical study	CBCT + periodontal eval.	Suture opening; skeletal/dental expansion; tipping; periodontal changes	T1–T2
Ning R, 2023 [37]	Prospective comparative clinical study	CBCT	Suture opening; skeletal/dental expansion; tipping; appliance comparison	T1–T2
Yoon A, 2022 [38]	Retrospective clinical analysis	Panoramic/CBCT	Complications; miniscrew failure; periodontal effects; adverse events	Post-treatment (retrospective)

CBCT = cone-beam computed tomography; 3D = digital model analysis/stereophotogrammetry; Clinical = evaluation without imaging (periodontal probing, soft-tissue exam, complications); T1 = pre-expansion; T2 = post-expansion; T3 = follow-up; Comparative = study comparing groups (e.g., age, appliance type, miniscrew length).

**Table 5:** Descriptive summary of the main clinically relevant adverse outcomes.

Outcome	N. of Studies Reporting the Outcome	Approximate n. of Patients Assessed	Reported Range/Proportion	Assessment Method
Alveolar bone loss/thinning	11/20 studies	758 patients *	Ranged from mild thinning to more pronounced reductions, depending on anatomical site and measurement method.	CBCT, periodontal examination
Buccal alveolar bone dehiscence	3/20 studies	Not consistently reported	Presence reported; quantitative prevalence varied across studies	CBCT, clinical examination
Failure of mid-palatal suture opening	6/20 studies	Not consistently Reported	Proportion of patients with expansion failure varied across studies	CBCT, radiographic assessment

* The total number of patients reflects the overall sample size of the included studies; not all outcomes were assessed in all patients.

**Table 6:** Technical findings of MARPE.

Study	Protocol of Expansion	Holding Period
Choi, 2016 [19]	t¼ turn activation (0.2 mm) every other day	3 months
Lim, 2017 [20]	¼ turn activation (0.2 mm) once a day	4 months
Bud ES, 2021 [21]	4/6 activations per day until the interincisor diastema opens, after 2 activations per day.	2 months
Madeeh S, 2023 [22]	N/R	N/R
Marin CM, 2023 [23]	1/4 turn in the morning and 1/4 turn in the evening	4 months
Park, 2017 [24]	1 turn activation per day	N/R
Clement, 2017 [25]	2 turn activation per day	4 months
Ngan, 2018 [26]	N/R	N/R
Shin, 2019 [27]	1 turn activation per day	N/R
Annarumma F, 2021	2 turn activation per day	12 months
* Lee, 2020 [29]	1 turn activation per day	N/R
Basu, 2023 [30]	After application it was activated by 1/4 turn. Then 2 turns per day until the diastema appears. After that, 1 turn per day.	N/R
Choi, 2023 [31]	¼ turn activation per day	3 months
Wang, 2023 [32]	1/2 activation per day until the diastema appears, later 1/4 turn.	6 months
Gokturk M, 2024 [33]	N/R	N/R
Kapetanovic A, 2022 [34]	¼ turn (0.25 mm) once a day	3 months
Calil, 2020 [35]	2/4 turn activation per day	4 months
Naveda R, 2023 [36]	1/4 turn activation per day twice a day	N/R
Ning R, 2023 [37]	N/R	6 months
Yoon A, 2022 [38]	N/R	4/6 months

* N/R = Not Reported; activation protocol and retention/holding period data were not available in all studies.

**Table 7:** Risk of bias summary for randomized studies (RoB 2).

Study	Study Bias from Randomization Process	Bias Due to Deviations from Intended Interventions	Bias Due to Missing Outcome Data	Bias in Measurement of the Outcomes	Bias in Selection of the Reported Result	Overall Rick of Bias
Basu, 2023 [30]	Low	Low	Moderate	Low	Some concern	Moderate
Choi, 2023 [31]	Low	Low	Some concern	Low	Some concern	Moderate

“Low” = low risk of bias; “Some concern” = some concerns regarding risk of bias; “Moderate” = moderate risk of bias.

**Table 8:** Risk of bias summary for non-randomized studies (ROBINS-I).

Study	Bias Due to Confounding	Bias in Selection of Participants	Bias in Classification of Interventions	Bias Due to Deviations from Intended Interventions	Bias Due to Missing Data	Bias in Measurement of Outcomes	Bias in Selection of the Reported Results	Overall Risk of Bias Judgment
Choi, 2016 [19]	Low	Low	NI	Low	NI	Moderate	Moderate	Serious
Lim, 2017 [20]	Low	Low	Low	Low	NI	Low	Moderate	Serious
Bud ES, 2021 [21]	Low	Low	NI	Low	Low	Moderate	Moderate	Serious
Madeeh S, 2023 [22]	Low	Low	NI	Low	Moderate	Moderate	Moderate	Serious
Marin CM, 2023 [23]	Low	Low	NI	Low	Moderate	Moderate	Moderate	Serious
Park, 2017 [24]	Low	Low	NI	Low	NI	Moderate	Moderate	Serious
Clement, 2017 [25]	Low	Low	NI	Low	Low	Moderate	Moderate	Serious
Ngan, 2018 [26]	Low	Low	NI	Low	Low	Moderate	Moderate	Serious
Shin, 2019 [27]	Low	Low	Low	Low	NI	Moderate	Moderate	Serious
Annarumma F, 2021 [28]	Low	Low	NI	Low	Moderate	Moderate	Moderate	Serious
Lee, 2020 [29]	Low	Low	NI	Low	NI	Moderate	Moderate	Serious
Wang, 2023 [32]	Low	Low	NI	Low	Moderate	Moderate	Moderate	Serious
Gokturk M, 2024 [33]	Low	Low	NI	Low	Low	Moderate	Moderate	Serious
Kapetanovic A, 2022 [34]	Low	Low	Low	Low	Low	Moderate	Moderate	Serious
Calil, 2020 [35]	Low	Low	NI	Low	Moderate	Moderate	Moderate	Serious
Nevada R, 2023 [36]	Low	Low	NI	Low	NI	Moderate	Moderate	Serious
Ning R, 2023 [37]	Low	Low	NI	Low	Moderate	Moderate	Moderate	Serious
Yoon A, 2022 [38]	Low	Low	Low	Low	Low	Moderate	Moderate	Moderate

NI = No Information; “Low” = low risk of bias; “Moderate” = moderate risk of bias; “Serious” = serious risk of bias. Risk of bias was assessed using the ROBINS-I tool for non-randomized studies.

**Table 9:** Grading of Recommendation, Assessment, Development, and Evaluation (GRADE) analysis.

Outcome	No. of Studies (Patients)	Assessment Methods	Main Limitations	Certainty of Evidence (GRADE)
Alveolar bone loss/thinning	11 studies (~758 patients *)	CBCT, periodontal examination	Serious risk of bias, high heterogeneity, limited or absent follow-up	⬤◯◯◯ Very low
Buccal alveolar bone dehiscence	3 studies	CBCT, clinical examination	Very small sample sizes, serious imprecision	⬤◯◯◯ Very low
Failure of mid-palatal suture opening	6 studies	CBCT, radiographic assessment	Observational design, inconsistency, non-standardized follow-up	⬤◯◯◯ Very low
Periodontal and gingival adverse events	5 studies	Clinical and periodontal examination	Heterogeneous outcome definitions, reporting bias	⬤◯◯◯ Very low
Device-related adverse events	2 studies	Clinical examination	Very limited data, rare events, serious imprecision	⬤◯◯◯ Very low

* The total number of patients reflects the overall sample size of the included studies; not all outcomes were assessed in all patients. ⬤ = high certainty, ◯ = low certainty. Certainty of evidence was assessed using the GRADE approach, considering risk of bias, inconsistency, indirectness, imprecision, and publication bias.

## Data Availability

No new data were created or analyzed in this study. Data sharing is not applicable to this article.

## Supplementary Materials

The following supporting information can be downloaded at: https://www.mdpi.com/article/10.3390/diagnostics16020352/s1, Table S1: PRISMA 2020 Checklist.
